# Gender- and Age-Related Changes in the Spectrum of the Differentiation Potential of Individual Colonies of Stromal Precursors from Human Bone Marrow

**DOI:** 10.3390/ijms27188414

**Published:** 2026-09-21

**Authors:** Irina Shipounova, Alena Dorofeeva, Tatiana Savvateeva, Kseniya Nikiforova, Nikolay Kapranov, Yuliya Davydova, Irina Galtseva, Anastasia Naumova, Alexandra Paderina, Olga Pokrovskaya, Larisa Kuzmina, Elena Parovichnikova

**Affiliations:** 1 National Medical Research Center for Hematology, 125167 Moscow, Russia; 2 Faculty of Biology, Moscow State University, 119991 Moscow, Russia

**Keywords:** mesenchymal stromal cells, human bone marrow, colony-forming units of fibroblasts, differentiation potential, bipotent precursors, osteogenic differentiation, adipogenic differentiation, gene expression, real-time PCR

## Abstract

Bone marrow (BM) mesenchymal stromal progenitors that give rise to almost all mature cells in the hematopoietic niche were studied. We analyzed the differentiation spectrum of individual colony-forming units of fibroblast (CFU-F) clones depending on the age and gender of the donor. Individual CFU-F clones (n = 292) were obtained from the BM samples of 19 donors (11 men, 8 women). The concentration of monopotent adipogenic precursors in the BM of men was significantly higher than that in the BM of women (15% ± 5% versus 4% ± 2%). Moreover, the concentration of monopotent adipogenic precursors in women’s BM had a clear negative correlation with age (R = −0.74), while, in men, it did not (R = −0.05). Younger donors of both genders had a higher proportion of monopotent adipogenic precursors; the proportion of precursors unable to differentiate increased markedly with age. All types of precursors revealed age- and gender-associated changes in the expression level of several genes. Both age and gender should be taken into account in studies concerning human stromal cells’ regenerative abilities. Alterations in the stromal precursors’ spectrum may impact the cellular composition of the bone marrow stromal microenvironment and explain the changes occurring in the regenerative potential with age and gender.

## 1. Introduction

In healthy human bone marrow (BM), the normal function of hematopoietic stem cells is performed by cells in hematopoietic niches. The niche, a specific microenvironment for HSCs, consists of stromal cells, the extracellular matrix, and soluble factors. In our work, we studied mesenchymal stromal progenitors, i.e., the cells that give rise to almost all mature cells in the stromal microenvironment. Stem/progenitor stromal cells keep the complex BM tissue safe and regenerate it throughout its lifespan. This includes renewing and remodeling the skeletal–muscular system and hematopoietic microenvironment, thereby healing fractures. Functionally, two types of stromal precursors can be analyzed in vitro, i.e., multipotent mesenchymal stromal cells and clonogenic stromal progenitors, which are identified by the colony-forming unit–fibroblast (CFU-F) assay. Mesenchymal stem cells are stromal cells that adhere to plastic and are capable of proliferation in culture, as well as differentiation in the osteogenic, adipogenic, and chondrogenic directions [1]. These cells were initially isolated from BM; however, today, researchers manage to obtain mesenchymal stem cells from a number of tissues [2]. As these cells do not entirely meet the criteria for stemness, they were proposed to be called multipotent mesenchymal stromal cells, and another criterion was added—they must express CD105, CD73, and CD90 on their surface and not CD45, CD34, CD14, CD11b, CD79a, CD19, or HLA-DR [2]. The NES+ cell population has been shown to include multipotent mesenchymal stem/stromal cells [3]. Mesenchymal stromal cells are a very popular topic of stromal cell differentiation and regeneration studies. However, historically, these cells were not the first to be reported as stromal precursors in the BM. CFU-Fs were described by Friedenstein and colleagues more than 50 years ago [4,5]. They defined the non-hematopoietic clonogenic cells in the bone marrow and showed that CFU-Fs possess a high but limited proliferative potential and can differentiate into bone, fat and cartilage. CFU-Fs are the more differentiated progeny of mesenchymal multipotent stromal cells [6]. Their differentiation abilities and multipotency have been analyzed in very few studies [7,8,9]. Donor gender had never been taken into account; the number of donors was also scarce. The only study where the age of the donors was addressed included five donors from 5 months to 30 years old [8]; thus, kids and young adults were analyzed. However, the regeneration of skeletal tissue is of high importance not only for children. On the contrary, older people have more problems with tissue remodeling. Bone remodeling is known to be tightly regulated by sex hormones [10,11]; however, the impact of the gender of the donor on the differentiation pattern of mesenchymal precursor cells has never been studied.

The aim of the work is to analyze the variation in the differentiation spectrum of individual CFU-F clones depending on the age and gender of the donor.

## 2. Results

### 2.1. Proliferative Abilities of Individual CFU-F Clones

We analyzed 19 BM samples from 11 male and 8 female donors. Only wells with a single clone were taken into account and counted as one clone for further calculations. The mean number of CFU-F colonies, obtained from one donor, was 36.7 ± 9.3. The difference between genders was insignificant (46.2 ± 14.7 for male BM samples vs. 30.2 ± 11.3 for female ones). There was no correlation between the number of clones and donor age (Pearson’s correlation coefficient R = −0.2). Each clone tested for its differentiation abilities originated from a single cell and grew to cover the area of three wells of a six-well cultivation plate, which is equal to 28.8 cm^2^. Such cell expansion indicated that these clones had a high proliferative capacity. The majority of clones (75% ± 5%) demonstrated a high proliferative potential and successfully survived two passages prior to the induction of differentiation. The mean time from BM cell plating to differentiation induction was 32 ± 1.1 days. In total, 292 clones were analyzed for their abilities to differentiate toward osteogenic and adipogenic lineages after the addition of corresponding inducers. Chondrogenic differentiation was not assessed due to technical limitations.

### 2.2. Differentiation Potential of Individual CFU-F Clones

**Figure 1:**
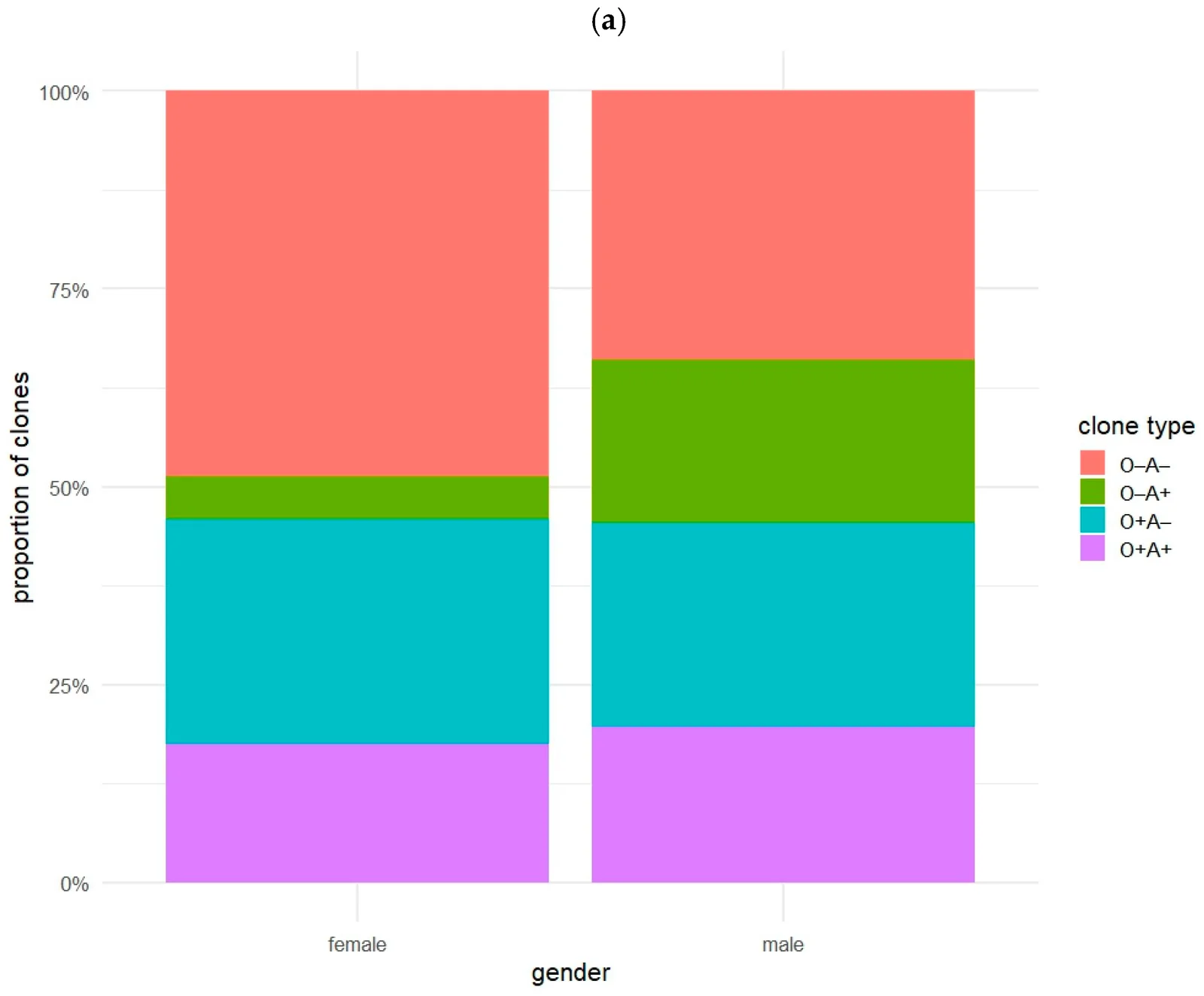

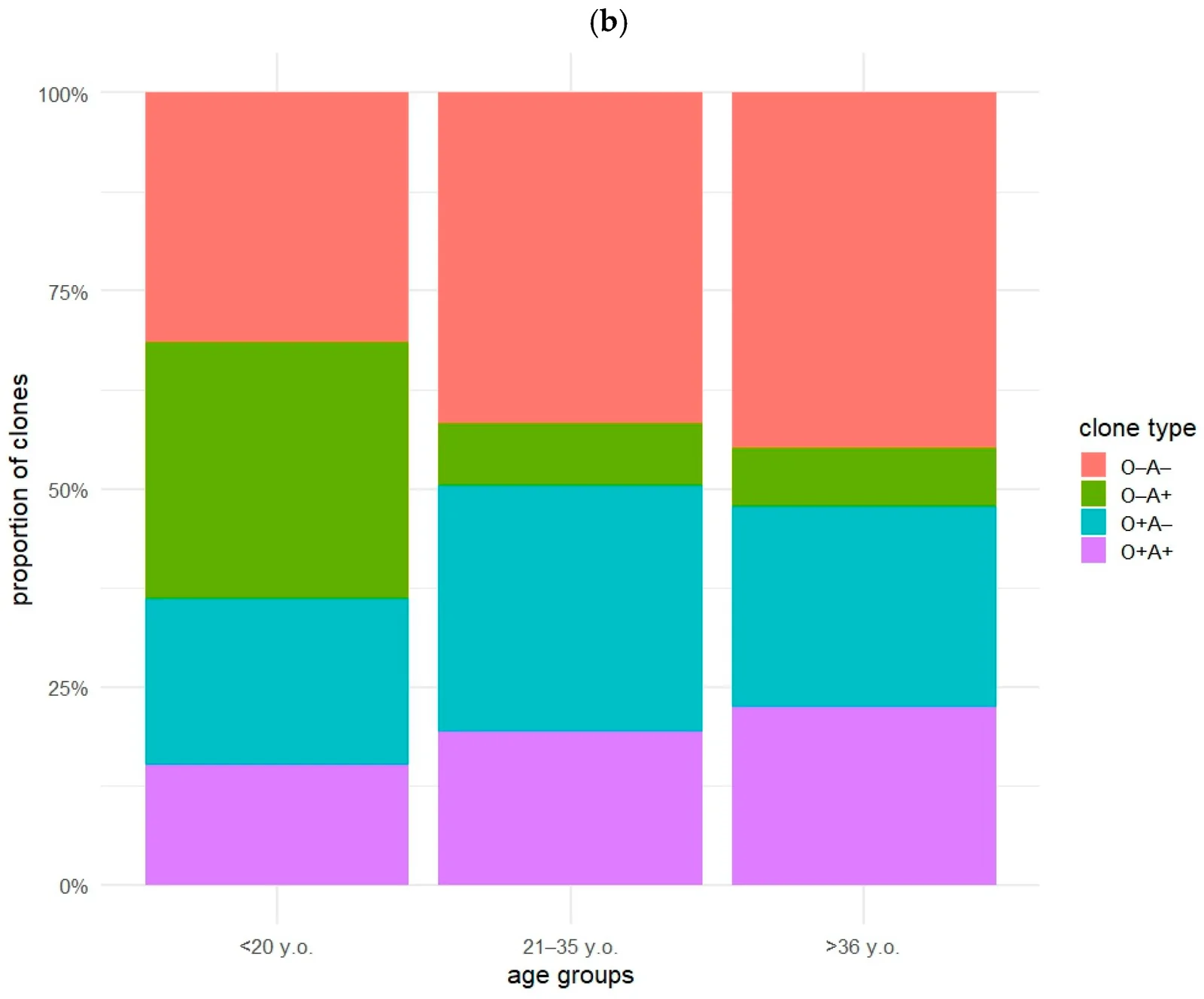
Distribution of clones with different differentiation potential (**a**) depending on donor’s gender—Fisher’s exact test, *p* = 0.01; and (**b**) depending on donor’s age—ANOVA between age groups revealed no statistically significant differences.

We classified all analyzed clones according to their ability to differentiate after the stimulation with corresponding inducers. The experimental design included only 2D cultivation; therefore, each clone was analyzed for its ability to differentiate toward the osteogenic and adipogenic lineages, but not toward the chondrogenic lineage. Clones with cells successfully differentiated toward both the osteogenic and adipogenic lineages were considered to be bipotent stromal precursors (indicated as O+A+, n = 55). Clones that differentiated toward only one lineage were considered monopotent osteogenic or adipogenic precursors (O+A−, n = 78, and O−A+, n = 44, respectively). Clones that failed to differentiate in either direction were considered to be fibroblast-like cells with a high proliferative potential, unable to differentiate toward the osteogenic or adipogenic lineages (O−A−, n = 115). An analysis of the differentiation potential of individual CFU-F clones from the donor BM revealed the heterogeneity of the CFU-F population. The distribution of clones with different potentials, depending on the gender and age of donors (with the impact of each donor taken into account), is presented in Figure 1.

When comparing the CFU-F clones isolated from the BM of men and women (Figure 1a), it turned out that the percentage of monopotent adipogenic stromal precursors in the BM of men was significantly higher (15% ± 5% versus 4% ± 2%; Fisher’s exact test, *p* = 0.01). The effect size, assessed using Cramér’s V, was 0.22 (weak–moderate level). When assessing differences in the proportions of clone types between men and women using linear models for each proportion separately, no statistically significant differences were found (all *p* > 0.05). A trend was observed toward a higher proportion of the O−A+ type in men compared to women (difference estimate: +0.11, *p* = 0.067), but the effect did not reach the level of statistical significance. A permutational multivariate analysis of variance (PERMANOVA) did not reveal significant differences in the overall profile of the clone type proportions between sexes (*R*^2^ = 0.031, *F* = 0.545, *p* = 0.558; 999 permutations). Thus, sex is not associated with a pronounced redistribution of the clonal profile in the studied cohort. Given the small sample size (n = 19), the power of the tests is limited, and moderate effects may have gone undetected. To confirm or refute the observed trends, studies with larger sample sizes are required.

To analyze the age-dependency of the stromal precursor distribution, we divided the donor group into three subgroups: 20 years old and younger (n = 5, 88 clones), 21–35 years old (n = 8, 130 clones), and older than 35 years (n = 6, 74 clones). A one-way analysis of variance (ANOVA) was performed on the proportions of four clone types (O+A+, O+A−, O−A+, and O−A−) across three age groups (<20 years, 21–35 years, and >36 years). No statistically significant differences in the proportions were found for any of the clone types. The proportion of monopotent adipogenic precursors (O−A+) decreased with age; on the contrary, the proportion of fibroblast-like cells unable to differentiate toward the osteogenic or adipogenic lineages (O−A−) increased (Figure 1b).

**Figure 2:**
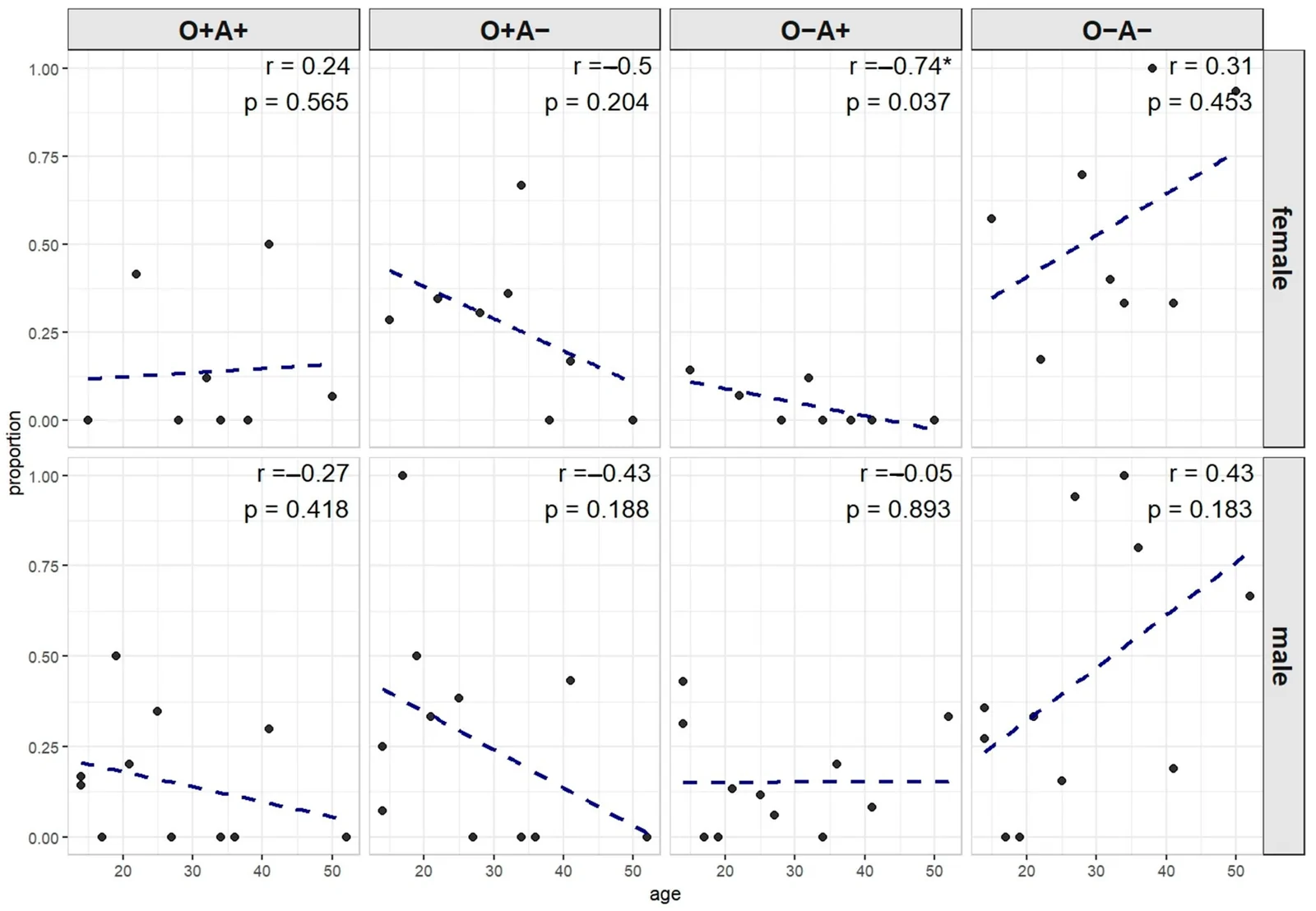
The relationship between the proportion of clone types and age in men and women (n = 19). Each panel corresponds to a separate combination of sex and clone type. The graph shows the observed values and the linear trend. The Spearman rank correlation coefficient (*r*) and the *p*-value are indicated in the upper right corner of each panel (* *p* < 0.05). Given the small sample size and multiple comparisons, the results should be interpreted as preliminary trends that require confirmation in an independent cohort.

The proportion of monopotent adipogenic precursors non-linearly varied between age groups. The cause of such an effect was suggested when the donor’s gender had been taken into account (Figure 2). The relationship between age and the proportions of the clone types was analyzed, taking into account the sample structure: each donor was a unit of observation (n = 19), and the proportions of clone types within each donor represented the compositional data (sum = 1). To assess the correlations, the Spearman rank correlation coefficient was used, separately in the subgroups of men and women and for each clone type. This approach allows for the correct accounting of the contribution of each donor and helps to avoid the incorrect aggregation of observations. Given the multiple tests (8 correlations) and the small subgroup sizes (n = 8–11), the results were considered as preliminary trends that require validation in an independent cohort.

It turned out that the proportion of monopotent adipogenic precursors in women’s BM had a clear negative correlation with age (R = −0.74), while, in men, there was no such correlation (R = −0.05) (Figure 2). The post hoc analysis of the correlation observed in females’ clones demonstrated a very high power of correlation (0.934). Therefore, the observed effect can be considered highly reliable.

In addition, we found controversial correlations in males’ and females’ O+A+ groups (Figure 2); however, the power of these correlations was low (0.24 for females and −0.27 for males, *p* > 0.05). The proportion of O+A− cells decreased with age for both genders, while the proportion of O−A− increased. The effect did not reach statistical difference.

### 2.3. Gene Expression in Individual CFU-F Clones with Various Differentiation Potential

Gene expression analysis for genes other than differentiation markers was performed on control (undifferentiated) cells for each clone’s triplicate (control cells; cells induced to osteogenic and to adipogenic differentiation). Based on the results of differentiation induction, each clone was assigned to one of four groups (O+A+, O+A−, O−A+, and O−A−), and gene expression data were aggregated according to the clone type.

Because only a limited amount of RNA was available for each clone, we focused on a small set of genes that could be analyzed using qPCR. The selected genes were *NES*, *IL10*, *ANGPT1*, *KITLG*, *VCAM1*, *TGFB1*, *FGFR1*, and *IL1B*. This gene set was chosen to evaluate the full range of key aspects of the stromal cell function in the hematopoietic microenvironment—from structural support and adhesion to the fine regulation of growth, inflammation, and blood cell maturation.

**Figure 3:**
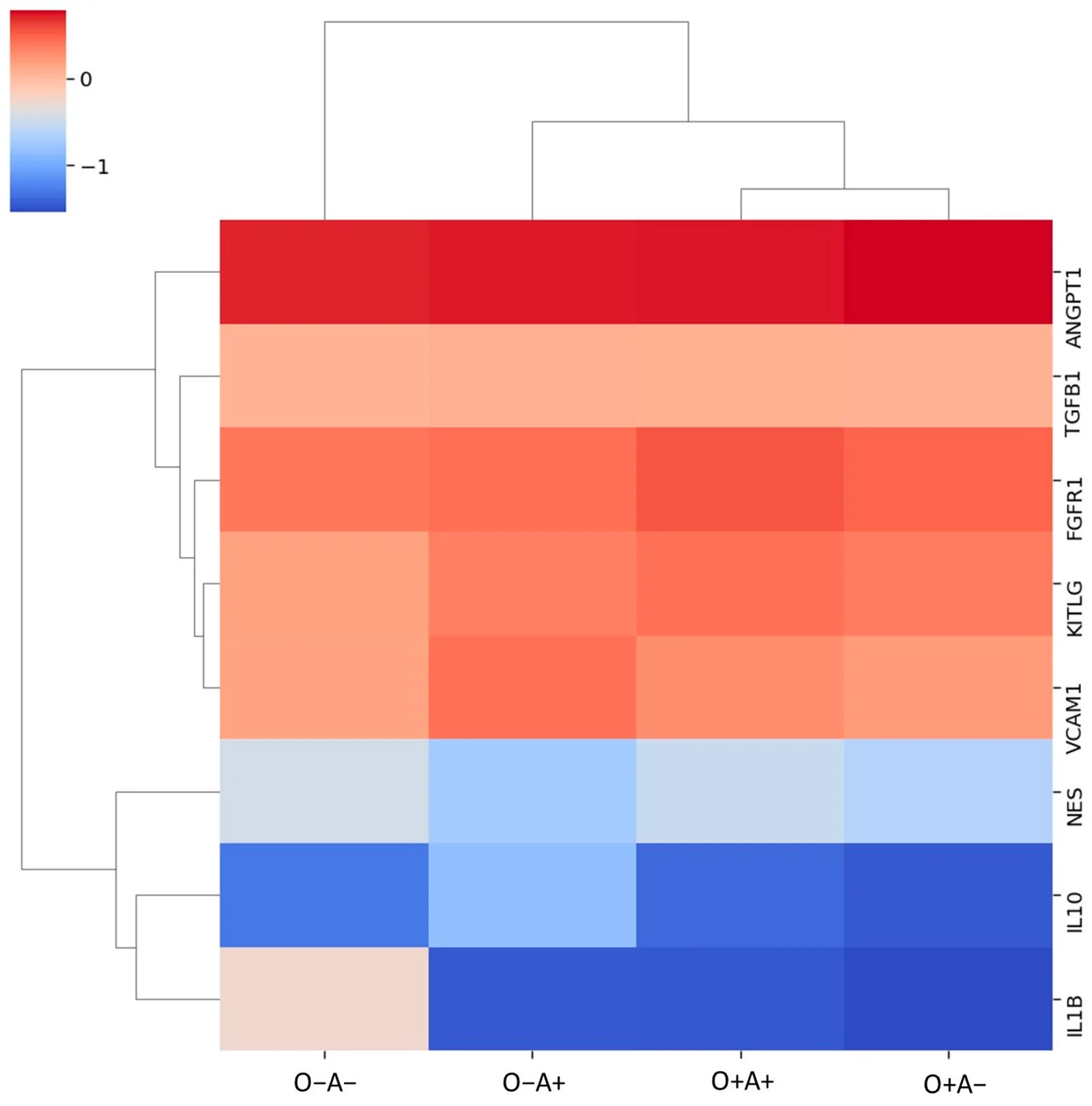
Heatmap and clustering analysis of expression levels of several genes in clones from all described groups.

A clustering analysis of gene expression across all clones revealed that the O+A+ group was the closest to the O+A− group, while the O−A− group was the farthest from all other groups (Figure 3).

Generally, in all types of stromal precursors, the relative expression levels (RELs) of *IL1B*, *IL10*, and *NES* were lower than those of *TGFB1*, *VCAM1*, *KITLG*, *FGFR1*, and *ANGPT1* (Figure 3).

**Figure 4:**
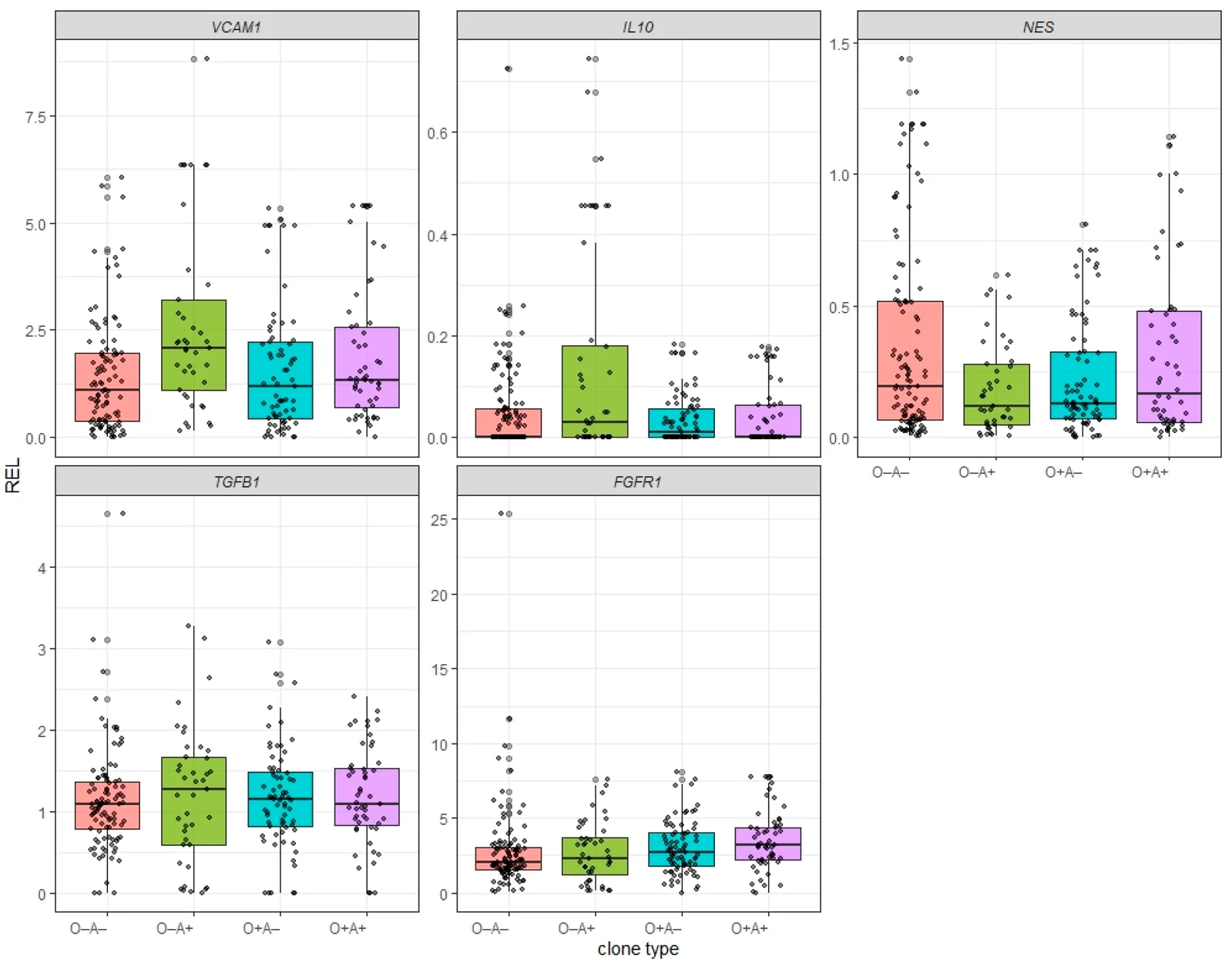
Relative expression level (REL) of differentially expressed genes. Benjamini–Hochberg multiple-testing correction was applied to all data.

Using the non-parametric Kruskal–Wallis test within each donor, followed by combining *p*-values using Fisher’s method, we identified significant differences in the expression between clone types for *VCAM1* (*p*_adj_ = 0.0008), *IL10* (*p*_adj_ = 0.0032), *NES* (*p*_adj_ = 0.036), and *TGFB1* (*p*_adj_ = 0.036). For the *FGFR1*, the differences were close to the significance threshold (*p*_adj_ = 0.048); for *KITLG* and *ANGPT1*, they slightly exceeded it (*p*_adj_ = 0.06). The analysis was based on data from 19 donors, which provided sufficient power to detect intergroup differences (Figure 4).

A linear mixed model was also applied to the gene expression data. According to the results of the linear mixed model with pairwise comparisons (Tukey), the expression of *VCAM1* differed significantly between clone types. The REL of *VCAM1* in the O−A+ group was significantly higher than in the O−A− group (*p* = 0.0045) and the O+A− group (*p* = 0.0139). The highest level of *IL10* gene expression was observed in the O−A+ group, and it differed significantly from the O−A−, O+A−, and O+A+ groups (*p* < 0.0001). Although there was a tendency towards differences, pairwise comparisons did not reveal statistically significant differences in the expression of the *NES*, *TGFB1*, and *FGFR1* between the clone types at the significance level of *p* < 0.05. The power of the interaction analysis is limited by the sample size (19 donors in the linear mixed model) and the uneven distribution of donors across subgroups; therefore, the absence of significant interaction does not rule out the presence of clinically important differences.

**Figure 5:**
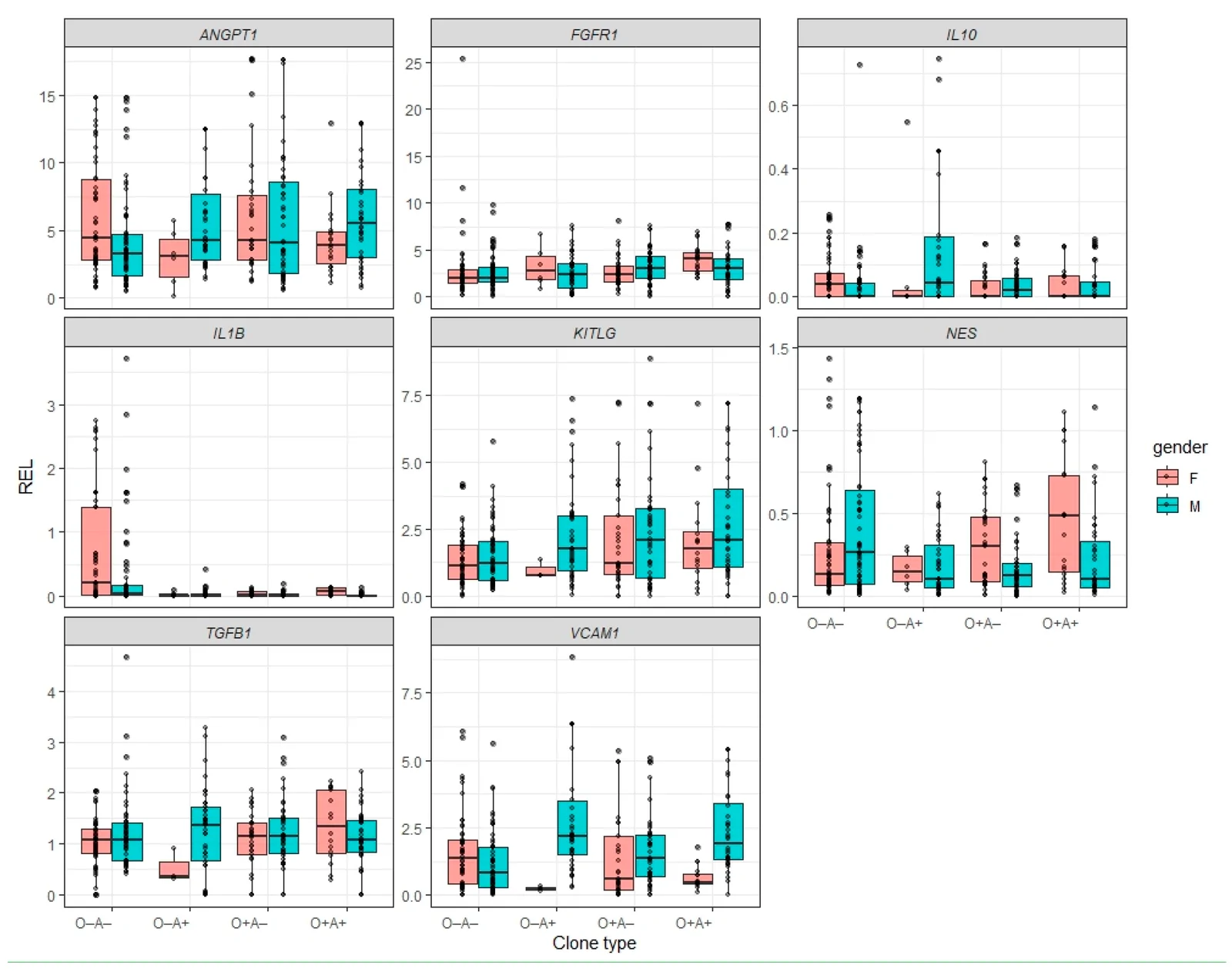
Relative expression level (REL) of differentially expressed genes, depending on the donor’s gender. F stands for female, M for male. Benjamini–Hochberg multiple-testing correction was applied to all data.

We then analyzed the gender-dependent differences in gene expression (Figure 5).

The most pronounced effects were observed for the *ANGPT1*, where significant positive trends were identified in two subgroups (O+A− in females, *p* = 0.005; O−A− in males, *p* = 0.020). The *NES* showed a significant decrease in expression only in women in the O+A− group (*p* = 0.0008). The *IL10* demonstrated a single significant effect in men in the O+A− group (*p* = 0.0002). The other genes did not show any significant associations with age in any subgroup.

**Figure 6:**
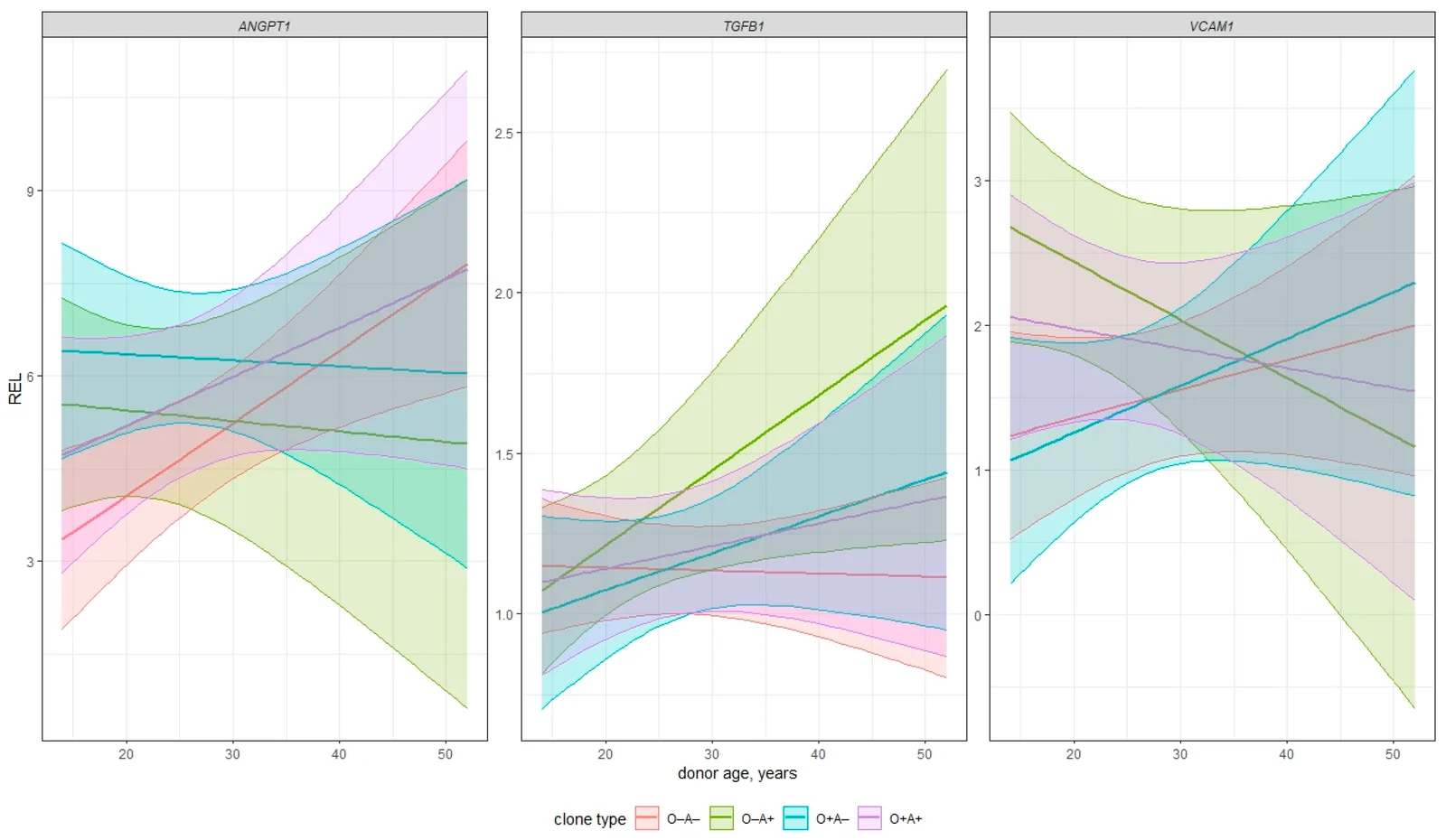
Relative expression level (REL) of differentially expressed genes, depending on the donor’s age.

To assess the relationship between gene expression and age in subgroups defined by the precursor type, linear mixed models with a fixed age effect and a random donor effect were used. The slope estimate (change in expression per 1 year of age) with the corresponding *p*-values was used as a measure of relationship. Additionally, for descriptive statistics, the Pearson correlation coefficient was provided for each subgroup. The expression levels of *VCAM1*, *TGFB1*, and *ANGPT1* were found to be statistically significantly changing with age in different CFU-F types (Figure 6).

For the *ANGPT1* gene, a significant linear effect of age was observed (*p* = 0.0055), with expression levels increasing on average with age. A significant association with the O+A− clone type was also detected (*p* = 0.041). Notably, within this subgroup, the expression decreased by 0.127 units per year, which contrasts with the overall positive trend. For the *VCAM1*, a significant interaction between the clone type and age was found (*p* = 0.041). Specifically, in the O−A+ type, the expression declined by an average of 0.06 units per year (95% CI does not include 0), whereas no statistically significant age-related change was observed for other clone types. A significant interaction between the O−A+ clone type and age was also identified for the *TGFB1* gene (*p* = 0.046). In this group, the expression increased by an average of 0.024 units per year.

## 3. Discussion

We analyzed individual CFU-F colonies derived from the BM of healthy donors. Approximately three-quarters of the clones exhibited a high proliferative potential, yielding a sufficient cell number for downstream analysis. We revealed that no significant correlation was observed between the donor age and number of clones. This finding may suggest that the pool of stromal precursors in the bone marrow remains stable within the examined age range (14–52 years).

Based on their differentiation characteristics, CFU-F clones with a high proliferative potential were classified into four subsets: bipotent osteogenic and adipogenic precursors, monopotent osteogenic precursors, monopotenr adipogenic precursors, and cells unresponsive to either induction medium. This classification confirms the heterogeneity of the CFU-F population. Notably, clonogenic stromal cells exhibiting a comparable proliferative capacity displayed distinct differentiation potentials.

The proportion of clones with osteogenic potential (O+A+ and O+A−) exceeded that of clones with adipogenic potential (O+A+ and O−A+). This pattern was consistent across both genders and all age groups within the studied cohort. These findings align with earlier reports by Merrywheather-Clarke et al. [7], Muraglia et al. [8], and Alessio et al. [9], which described a similar bias toward osteogenic precursors. However, those studies were limited by small donor numbers (three to five donors each) and largely did not account for donor sex or age (with the exception of Muraglia et al., who included five donors aged 5 months to 30 years). In contrast, our analysis encompassed BM stromal precursors from 19 donors spanning a broader age range (14–52 years), enabling a more robust assessment of age- and sex-related effects. The observed reduction in the frequency of adipogenic precursors was consistent across sexes and age groups, an effect likely undetected in prior studies due to their substantially smaller sample sizes.

No statistically significant differences in the proportions of stromal precursors across age groups were detected. A post hoc power analysis, based on the empirical effect sizes observed in the sample, indicated a low statistical power for the current study design. With an average sample size of ~7 donors per age group, the power to detect the identified effects ranged from 5% to 47%. This explains the lack of statistically significant differences and indicates the need to increase the sample size to reliably detect moderate effects. Despite the lack of statistical significance, a pronounced age-dependent trend was evident in the data. Specifically, a marked decrease in the proportions of monopotent adipogenic (O−A+) clones combined with an increase in the proportion of O−A− clones was demonstrated. Notably, more than half of fibroblastic colonies derived from the BM of donors older than 36 years exhibited a high proliferative potential but were unresponsible to the applied differentiation induction media. This pattern may at least partially explain the age-related decline in the regenerative capacity of the skeletal system. As we did not observe the correlation between donor age and total clone number, the impaired regeneration in older individuals is likely attributable to a loss of differentiation potential rather than a reduction in proliferative capacity. Further studies should investigate the differentiation and proliferative states of clones in the bone marrow of individuals older than 52 years.

It should be noted that the correlations for individual clone types are dependent on the compositional nature of the data: a change in the proportion of one clone type inevitably leads to changes in the proportions of other types. Therefore, the identified correlations should be considered as complementary characteristics of the clonal profile rather than as isolated effects. All analyses were stratified by sex to correctly account for the contribution of each donor and to increase the transparency of the interpretation.

Gene expression clustering analysis revealed that bipotent (O+A+) and monopotent osteogenic (O+A−) precursors exhibited a greater similarity to each other than monopotent adipogenic (O−A+) precursors or non-responsive (O−A−) clones. Monopotent adipogenic clones (O−A+) showed a closer similarity to the bipotent (O+A+) and monopotent osteogenic (O+A−) groups than to the O−A− subpopulation. Notably, O−A− clones were the most distinct from all other groups, suggesting a heterogeneous nature of O−A− population.

The cellular composition of the O−A− group can be inferred from the expression results. Within the O−A− group, a high and stable expression of *ANGPT1* and *KITLG* is observed—a characteristic of cells that support the vascular and hematopoietic niches (pericytes, MSCs, osteoblasts, and specialized stromal cells). The variable expression of *VCAM1*—from low to high—is typical for a heterogeneous stromal population that includes endothelial and activated mesenchymal cells. A moderate *NES* expression indicates the presence of cells with a mesenchymal/stem cell phenotype [12], but not a homogeneous stem cell population. A low *IL1B* and *IL10* expression suggests the absence of strong inflammatory activation, pointing to the fact that the population is predominantly quiescent or supportive. A stable expression of *FGFR1* and *TGFB1* is typical for mesenchymal and stromal cells involved in maintaining the extracellular matrix and regulating differentiation. We propose that the O−A− subpopulation comprises a heterogeneous stromal cell fraction, most likely including MSCs (they explain the expression of *NES*, *KITLG*, *ANGPT1*, *FGFR1*, and *TGFB1*), pericytes, and vascular smooth muscle cells (they explain the high levels of *ANGPT1* and *KITLG*, as well as the variable expression of *VCAM1*), and endothelial cells and their precursors (they explain the presence of *VCAM1* and *KITLG*, as well as the moderate expression of *NES* in some subpopulations). Importantly, the data include samples from different donors (different ages and genders), which explains the variability in expression levels. Younger donors may have a higher proportion of stem-like phenotypes, reflected by a higher *NES* expression [12]. Older donors may harbor a predominance of the differentiated stromal component (reflected in a higher *FGFR1* and *TGFB1* expression, and lower *NES* expression). This confirms our suggestion that O−A− clones do not represent a homogeneous cell population, but rather a heterogeneous stromal fraction that includes MSCs, pericytes, and endothelial cells in varying proportions.

Merrywheather-Clarke et al. [7] reported a weak positive correlation between osteogenic abilities and vascular tubes formation. We examined whether a similar correlation was evident in our data. In females, RELs of *VCAM1* and *ANGPT1* were downregulated in the monopotent osteogenic progenitors’ subpopulation. Moreover, the *VCAM1* expression was upregulated in the O−A+ subpopulation derived from younger donors. In contrast, other age groups and precursor types tended to demonstrate the age-associated downregulation of *VCAM1* expression. In contrast to these patterns, the REL of *ANGPT1* was generally higher in the monopotent O+A− stromal precursors regardless of gender. Age-related trends were also observed. On average, its expression increased with age across the cohort; yet, within the O+A− subgroup, a decrease was detected. This finding contrasts with the overall trend.

The coordination between osteogenesis and vascularization is of high importance for the regeneration of fully functional bone and bone marrow tissues. In our study, the two vasculogenesis-related genes (*ANGPT1* and *VCAM1*) demonstrated a possible age-dependent association with osteogenesis, particularly in monopotent osteogenic precursors. These results partially contradict the findings of others [7] and are demonstrated solely on the RNA-level measurements. Therefore, further investigation is needed. Future studies should also explore the possible role of adipogenic precursors in the regulation of vasculature formation. Further investigations of a wider range of angiogenic genes and proteins should answer this question.

Monopotent adipogenic O−A+ clones exhibited higher expression levels of *IL10* and *VCAM1* compared to other clone types. Given that both genes are negatively regulated by *GATA3* [13,14], this pattern suggests a potential downregulation of *GATA3* in adipogenic precursors. The expression level of *GATA3* was not assessed in the present study and will be addressed in future work.

The sample size allowed us to analyze males and females separately, revealing unique patterns. The proportion of monopotent adipogenic precursors (O−A+) was dramatically lower in BM samples from females compared to males. A detailed examination showed that, in males, the proportion of O−A+ precursors remained stable across age groups, whereas, in females, a clear age-related decline was observed, with a high correlation coefficient. One could suggest the more intensive exhaustion of the adipogenic precursor pool in the female BM over time.

In contrast, the proportion of monopotent osteogenic (O+A−) precursors decreased with age both in males and in females in a similar manner. The age-dependent increase in the proportion of O−A− clones was also similar for both genders, although the correlations were not statistically significant. The news is good as far as the regenerative perspective is concerned. We demonstrate that the osteogenic precursors remain present in a relatively high proportion in BM with ageing, and, importantly, they are completely functional. Thus, they could contribute to bone tissue formation if properly stimulated.

Sex-associated differences in gene expression were revealed across CFU-F subpopulations. The most pronounced effect was observed for the *ANGPT1*, which was increased in O+A− cells in females. In contrast, the *NES* expression was significantly reduced in the same clone type in females. Notably, the same CFU-F type in men demonstrated a distinct pattern, with significant *IL10* upregulation. These gender-dependent patters reveal that age-related gene expression changes differ between males and females, which is important for understanding the regenerative potential of stromal cells.

The donor’s gender is frequently overlooked in published works. While some investigators try to matched the control and experimental groups by age and sex, the majority of reports involve donor sample within a narrow age range and rarely provide information on the donor sex. Our work aims to draw attention to the gender- and age-associated heterogeneity in the BM stromal precursor population. The consideration of sex and age, in our view, is not only relevant for the practical aspects of research, but also critical for refining fundamental experimental frameworks and data interpretation.

## 4. Materials and Methods

Individual CFU-F clones (n = 292) were obtained from BM samples of 19 donors (8 women and 11 men, 14–52 years old, median 28 years). BM samples from healthy donors were obtained during BM donation for transplantation. All manipulations were conducted in accordance with the Declaration of Helsinki (1964). This study was approved by the local ethics committee; donors provided written informed consent.

**Figure 7:**
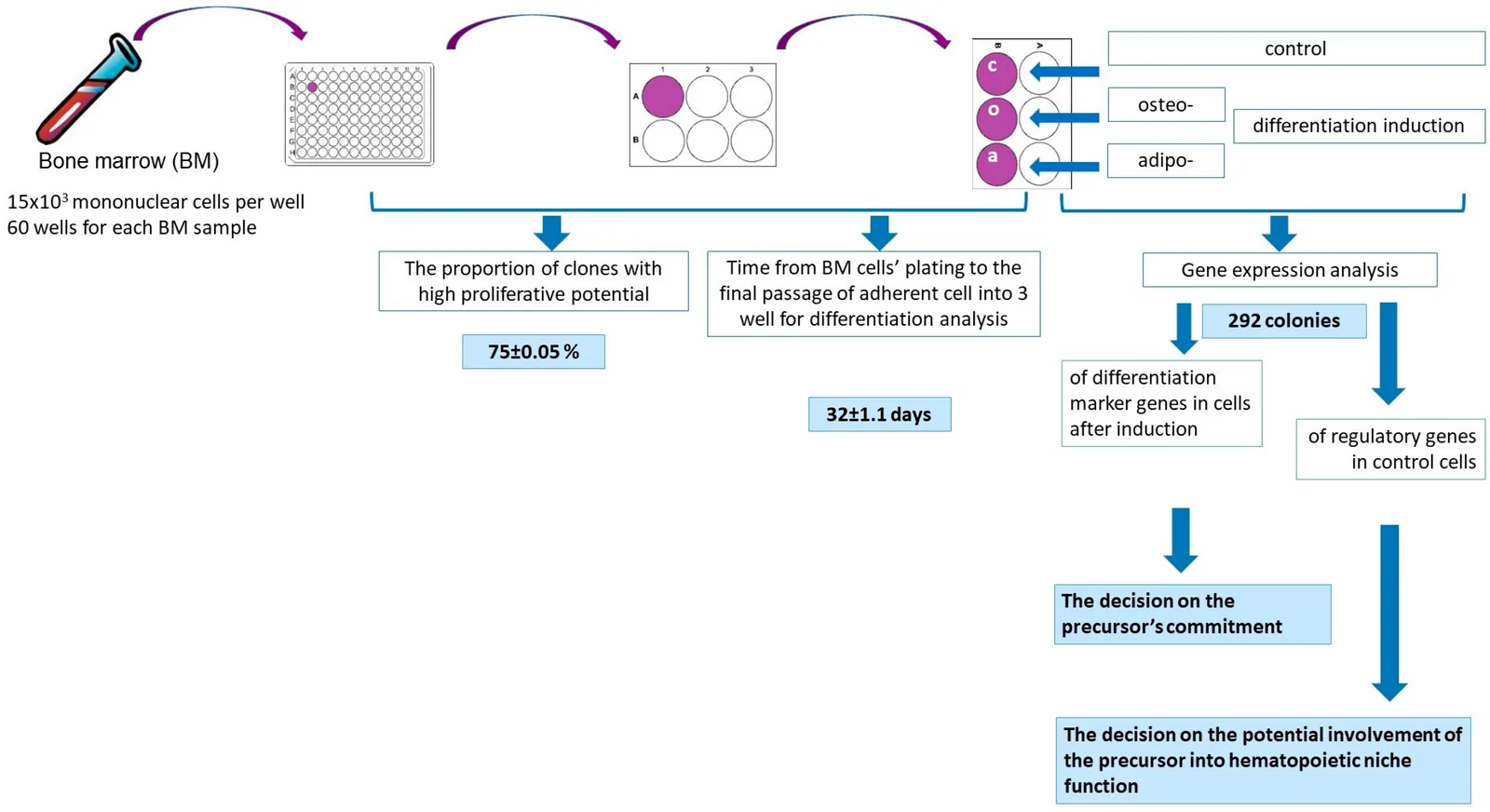
Experimental design. ‘C’ stands for ‘control’; ‘o’ for ‘osteogenic differentiation’; and ‘a’ for ‘adipogenic differentiation’.

The experimental design is presented on Figure 7.

The BM mononuclear cells were separated as follows. The aspirated BM cells were sterilely diluted with αMEM (ICN Biomedicals, Costa Mesa, CA, USA) medium containing 0.2% methylcellulose (density 1500 cP, Sigma-Aldrich, St. Louis, MO, USA) in a 1:1 ratio and left for 40 min at room temperature. During this time, most of the erythrocytes and granulocytes sedimented, while the mononuclear cells remained in suspension. The suspension was carefully collected, transferred to a sterile centrifuge tube, and sedimented by centrifugation at 1500 rpm for 10 min. The cell pellet was resuspended in the required volume of cultivation medium and used for further experiments. Each BM sample was analyzed for the following parameters: number of mononuclear cells per 1 mL of BM aspirate (16.8 ± 2.8 × 10^6^ cells/mL), the proportion of CD45+ cells (99.98 ± 0.003%), and the proportion of non-hematopoietic cells with CD45-CD34-CD71-CD235-CD90+CD73+CD105+ phenotype (0.023 ± 0.003%). Cell immunophenotype was determined by flow cytometry on BD FACS CANTO II (Becton Dickinson, Franklin Lakes, NJ, USA) after incubation with fluorescent-labeled monoclonal antibodies (all from Becton Dickinson, Franklin Lakes, NJ, USA). For each of the fluorochromes, an appropriate isotype control was used (all from Becton Dickinson, Franklin Lakes, New Jersey, USA). The data were processed using BDFACSDiva 8.0.2 software (BD Bioscience, Franklin Lakes, NJ, USA).

After separation, 15 × 10^3^ nucleated cells were plated per well of a 96-well plate in αMEM supplemented with 10% fetal bovine serum (HyClone, Logan, UT, USA), 2 mM L-glutamine (ICN Biomedicals, Costa Mesa, CA, USA), 100 U/mL penicillin (Sintez, Moscow, Russia), and 50 μg/mL streptomycin (BioPharmGarant, Vladimir, Russia). Cultures were carried out at 37 °C and 5% CO_2_. One plate with 60 seeded wells was prepared for each marrow sample. Cultures were examined biweekly for the appearance of stromal colonies. Wells containing more than one colony were not included in the analysis. Every clone that reached confluence was detached with 0.25% trypsin (ICN Biomedicals, Costa Mesa, CA, USA), in 0.02% EDTA (ICN Biomedicals, Costa Mesa, CA, USA), in 0.9% NaCl (Sigma-Aldrich, St. Louis, MO, USA), and plated in 1 well of a 6-well plate. After reaching confluence, the cells of each clone were divided into 3 wells assigned for control and for the induction of osteogenic and adipogenic differentiation. Osteogenic differentiation medium contained 0.1 μM dexamethasone (Sigma-Aldrich, St. Louis, MO, USA), 0.15 mM ascorbic acid 2-phosphate trisodium salt (ICN Biomedicals, Costa Mesa, CA, USA), and 3 mM NaH_2_PO_4_ (ICN Biomedicals, Costa Mesa, CA, USA). Adipogenic induction culture medium was supplemented with 1 μM dexamethasone (Sigma-Aldrich, St. Louis, MO, USA), 60 μM indomethacin (Sigma-Aldrich, St. Louis, MO, USA), and 5 μg/mL insulin (Sigma-Aldrich, St. Louis, MO, USA). The induction lasted for 2 weeks with changing cultivation media twice a week.

RNA was extracted from individual clones following completion of the induction protocol for subsequent gene expression analysis. Cells were washed twice with Dulbecco’s Phosphate Buffered Saline (MP Bio-medicals, Illkirch-Graffenstaden, France) and RNA were isolated by modified Chomczynski method [15] using TRIZOL (Ambion (Thermo Fisher Scientific), Austin, TX, USA). In subsequent reverse-transcription reaction, M-MLV reverse-transcriptase (Promega Corporation, Madison, WI, USA), and mixture of poly-T oligonucleotides and random hexamers in equal proportions as primers were used. Real-time polymerase chain reaction in TaqMan modification was carried out on 7500 amplificator (Applied Biosystems, Foster City, CA, USA). Target genes, as well as primers and probes sequences used, are listed in the Supplementary Materials (Table S1). Relative gene expression level was determined by ∆∆Ct method as described previously [16]. For normalization, *GAPDH* and *BACT* were used as reference genes.

**Figure 8:**
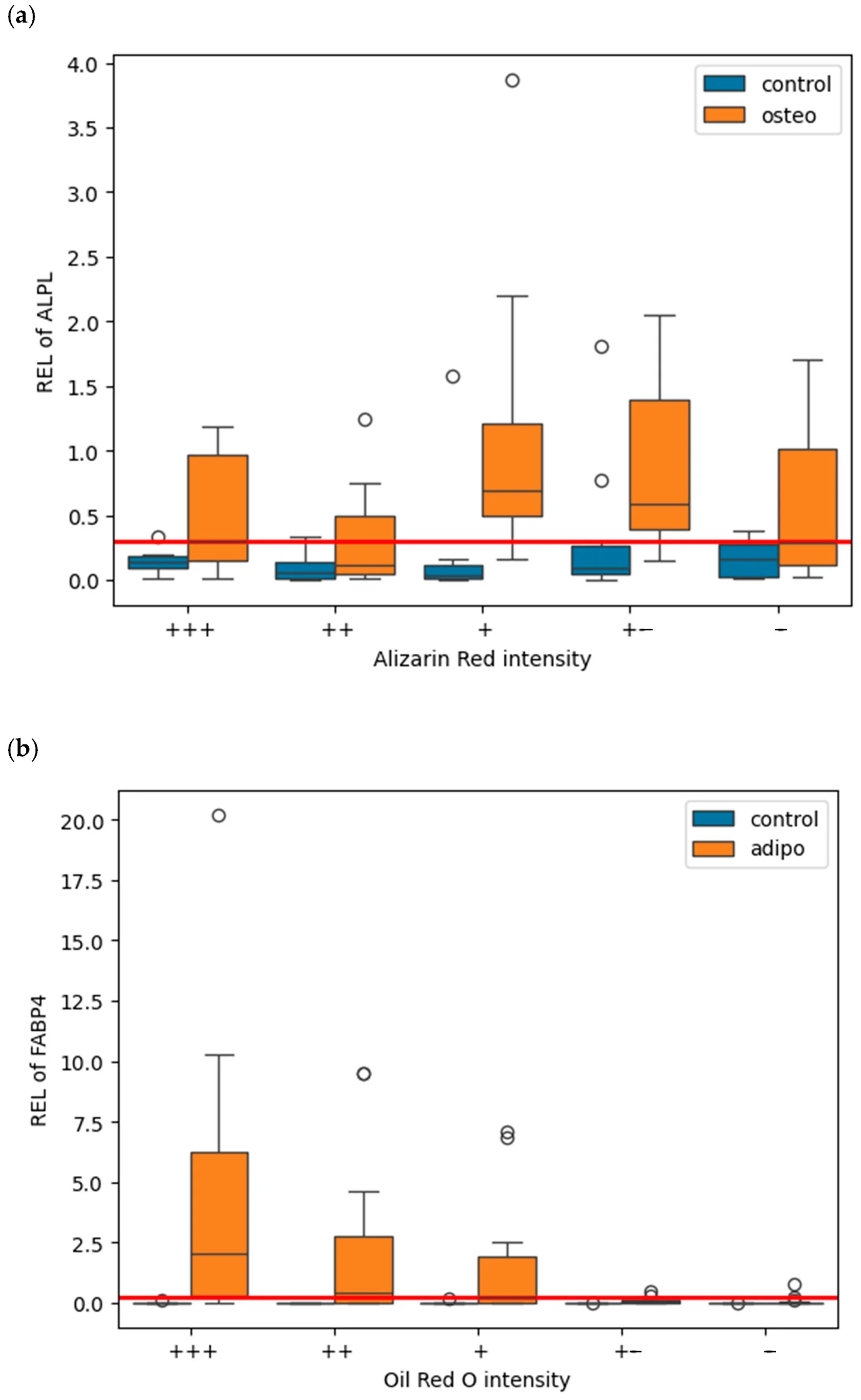
REL of expression of differentiation genes used for CFU-F type classification. (**a**) *ALPL* and (**b**) *FABP4*; the threshold was chosen arbitrary, aiming to cut out undifferentiated control cells. Staining intensity was estimated visually, ranging from ‘very intensive’ (+++) to ‘rarely visible’ (−).

Differentiation marker genes and expression thresholds were selected based on association of their expression level with the intensity of specific staining (Alizarin Red for osteogenic induction, and Oil Red O for adipogenic induction). The intensity of staining was estimated visually, ranging from ‘very intensive’ (+++) to ‘rarely visible’ (−). Twelve samples were chosen for each intensity group. Each sample was analyzed in duplicates—one well was stained, while another was analyzed for gene expression. We tested the expression levels of *PTHR1*, *ALPL*, and *RUNX2* as markers of osteogenic induction, and *PPARG*, *LEP*, and *FABP4* as markers of adipogenic induction. *ALPL* and *FABP* were chosen as the most suitable for distinguishing between control and differentiated cells (Figure 8a and Figure 8b, respectively).

**Figure 9:**
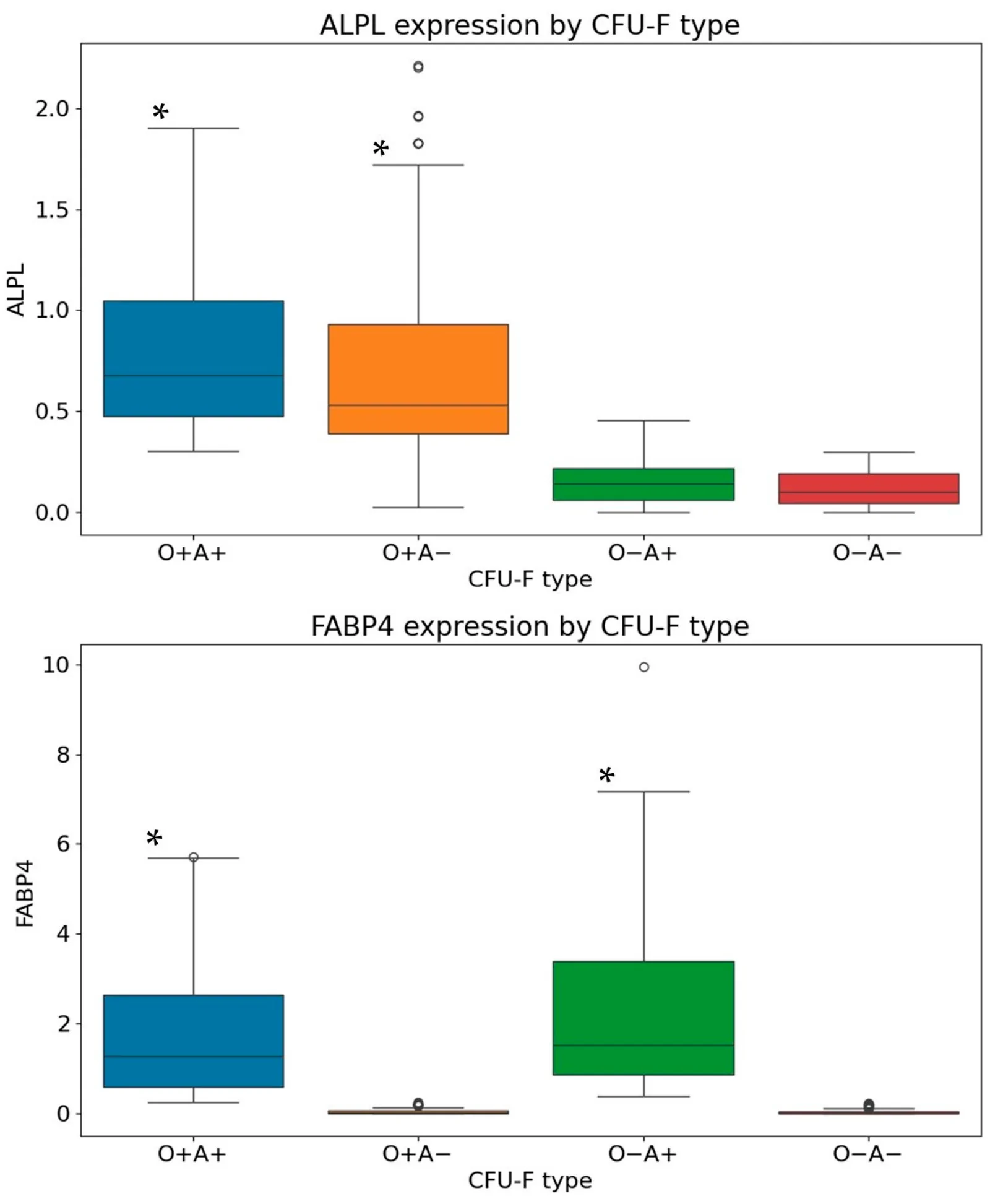
REL of expression of differentiation genes serving for CFU-F type distinguishing. O+A+ clones had high REL of *ALPL* and *FABP4*; O+A− had high REL of *ALPL* only; O−A+, of *FABP4* only; O−A− had REL of both genes on low level; * indicates significant differences between upregulated and downregulated gene expression estimated by ANOVA followed by Tukey’s HSD post hoc test.

Finally, the ability to differentiate was evaluated by changes in the expression level of *FABP4* (adipogenesis) and *ALPL* (osteogenesis) genes in all cells subjected to induction and their correspondent control cells. The criteria of subpopulation classification are visually presented on Figure 9. Briefly, all cells from each clone had been analyzed for the REL of *GAPDH* and *BACT*, *ALPL* (controls and after induction of osteogenesis), and *FABP4* (controls and after induction of adipogenesis). After normalization, the results for *ALPL* and *FABP4* expression for each subgroup (control, osteo-, and adipogenic induction) of each clone were analyzed. The expression level was marked as high or low based on the previously chosen thresholds. Clones with both high *ALPL* expression level in osteo- and high *FABP4* expression level in adipo-differentiation were defined as O+A+ clones. Clones with high *ALPL* expression level in osteo- and low *FABP4* expression level in adipo-differentiation were defined as O+A− clones. Clones with low *ALPL* expression level in osteo- and high *FABP4* expression level in adipo-differentiation were defined as O−A+ clones. Clones with both low *ALPL* expression level in osteo- and low *FABP4* expression level in adipo-differentiation were defined as O−A− clones.

The relative expression level of *NES*, *IL10*, *ANGPT1*, *KITLG*, *VCAM1*, *TGFB1*, *FGFR1*, and *IL1B* was evaluated in the control undifferentiated cells of the clones. Benjamini–Hochberg multiple-testing correction was applied to all data.

The data are presented as mean ± SEM. The significance of the differences was determined by the ANOVA followed by Tukey’s HSD post hoc test or the Mann–Whitney U-test. The differences were considered significant at *p* < 0.05. The comparison of the precursor type distribution with age or gender was performed with Chi-square test. The correlation with age was assessed by the Pearson correlation coefficient. Comparison of two independent Pearson correlation coefficients was done using Fisher’s r-to-z transformation. The sample size (n = 19 donors) was determined by the available samples; the analysis power to detect interactions was limited, which was taken into account when interpreting the absence of significant effects.

The analysis of differential gene expression was performed using linear mixed-effects models in the R environment (version 4.6.1). The fixed effects included the clone type (CFU-F_type), the donor’s gender (gender), or the donor’s age (age), as well as their interactions; the random effect was the donor identifier (donor_ID) to account for the intra-donor correlation of clones. Models were built using the ‘lme4’ package (lmer function); the significance of the fixed effects was assessed using the ‘lmerTest’ package (Satterthwaite approximation of degrees of freedom).

To assess the main effects and interactions, we calculated model coefficients, standard errors, t-statistics, and the corresponding *p*-values. To interpret the interactions and construct predicted mean values by category, we used the ‘emmeans’ package. In particular, to analyze the dependence of expression on age, predicted means were constructed on a uniform age grid (from the minimum to the maximum value in the sample, 50 points) using the emmeans (~CFUf_type * age, at = list(age = …)) function. To estimate the rate of change in expression with age (trend slope), the emtrends (mod, ~CFUf_type, var = “age”) function was used, obtaining estimates of the change in expression per 1 year of age for each clone type separately.

The results were visualized in Python 3 using Pandas, Matplotlib, and Seaborn libraries, and in RStudio 2026.01.2 using the ‘ggplot2’ and ‘dplyr’ packages. For genes in which the model demonstrated a singular boundary (singular fit), the text of the results and limitations included a corresponding caveat about the potential underestimation of standard errors.

The analysis of the effect of donor gender on gene expression was carried out within the framework of separate linear mixed models that included clone type, gender, and their interaction as fixed effects, and donor identifier as a random effect. The mean values and confidence intervals for the “clone type × gender” combinations were calculated using emmeans.

All statistical analyses were created in RStudio 2026.01.2. using the abovementioned R packages and in Python 3 using Pandas, Matplotlib, Seaborn, Scikit-learn, and SciPy libraries.

## 5. Conclusions

In summary, age-associated declines in the proportion of monopotent adipogenic precursors were observed in female BM. The proportion of monopotent osteogenic precursors decrease with age in BM samples from men and women. The proportion of BM progenitors that do not respond to osteogenic or adipogenic differentiation induction increased with age, regardless of gender. Furthermore, distinct age- and gender-associated patterns of gene expression were observed across different types of stromal precursors.

We found age- and gender-associated differences in the stromal precursor compartment of human bone marrow. These characteristics should be explicitly accounted for in studies evaluating the regenerative abilities of stromal cells. Alterations in the spectrum of stromal precursors may affect the cellular composition of the bone marrow stromal microenvironment. This, in turn, may contribute to the changes occurring in the regenerative potential with age and gender.

## Data Availability

The data presented in this study are available from the corresponding author upon reasonable request.

## Supplementary Materials

The following supporting information can be downloaded at: https://www.mdpi.com/article/10.3390/ijms27188414/s1.

## Abbreviations

The following abbreviations are used in this manuscript:

BM	Bone marrow
CFU-F	Colony-forming units of fibroblasts
REL	Relative expression level
O+A+	Bipotent osteo- and adipogenic precursor cells
O+A−	Monopotent osteogenic precursor cells
O−A+	Monopotent adipogenic precursor cells
O−A−	Fibroblast-like cells with high proliferative potential, failed to differentiate toward osteogenic and adipogenic lineages
