# Supplementary material for: Gender- and Age-Related Changes in the Spectrum of the Differentiation Potential of Individual Colonies of Stromal Precursors from Human Bone Marrow
Source: Int J Mol Sci. 2026 Sep 21;27(18):8414. doi: 10.3390/ijms27188414 (PMC13607302; doi:10.3390/ijms27188414)
Supplement: Supplementary file 1 [file ijms-27-08414-s001.zip › Supplement.pdf]

Table S1. Primers and probes for TaqMan real-time PCR, designed by authors and used in the study

gene		sequence
ALPL	forward	GGTCACCTCCATCCTGCG
	reverse	CATCTCGTTGTCTGAGTACC
	probe	FAM- CAATGCCCACAGATTTCCCAGCGTCCT -RTQ1
ANGPT1	forward	CAGTCAGAGGCAGTACATGCTAAG
	reverse	TTCCTGCTGTCCCAGTGTGA
	probe	FAM-TGAATAGGCTCGGTTCCCTTCCCAGT-RTQ1
BACT	forward	CAACCGCGAGAAGATGACC
	reverse	CAGAGGCGTACAGGGATAGC
	probe	ROX-AGACCTTCAACACCCCAGCCATGTACG-BHQ2
FABP4	forward	ATGATAAACTGGTGGTGGAAT
	reverse	TCAATGCGAACTTCAGTCC
	probe	FAM-TGGCTTATGCTCTCTCATAAACTCTCG-RTQ1
FGFR1	forward	CAGAATTGGAGGCTACAAGG
	reverse	TGATGCTGCCGTACTCATTC
	probe	FAM- CATCATAATGGACTCTGTGGTGC-RTQ1
GAPDH	forward	GGTGAAGGTCGGAGTCAACG
	reverse	TGGGTGGAATCATATTGGAACA
	probe	ROX-CTCTGGTAAAGTGGATATTGTTGCCATCA-BHQ2
IL1B	forward	ATTCTCTTCAGCCAATCTTCA
	reverse	AAGGAGCACTTCATCTGTTTA
	probe	FAM-AGAACAAGTCATCCTCATTGCCAC-RTQ1
IL10	forward	CCGTGGAGCAGGTGAAGA
	reverse	TTGTCATGTAGGCTTCTATGTAGT
	probe	FAM-ATAAGCTCCAAGAGAAAGGCATCTAC-RTQ1
KITLG	forward	TCTGCAGGAATCGTGTGACT
	reverse	TCTCGCTTATCCAACAATGAC
	probe	FAM-CTTCCAAAAGACTACATGATAACC-RTQ1
NES	forward	ACCTCAAGATGTCCCTCAGC
	reverse	GGGAGCAAAGATCCAAGACG
	probe	FAM-CTCCAAGACTTCCCTCAGCTTTCAG-RTQ1
TGFB1	forward	TGCGTCTGCTGAGGCTCAA
	reverse	CGGTGACATCAAAAGATAACC
	probe	FAM-AGGAATTGTTGCTGTATTTCTGGTAC-RTQ1
VCAM1	forward	CCGAAAGGCCCAGTTGAAG
	reverse	CACGAGAAGCTCAGGAGAAA
	probe	FAM-AACAAAGTTGGCTCACAATTAAGAAGTTT-RTQ1
